# The parasite cytokine mimic *Hp*‐TGM potently replicates the regulatory effects of TGF‐β on murine CD4^+^ T cells

**DOI:** 10.1111/imcb.12479

**Published:** 2021-07-01

**Authors:** Madeleine P J White, Danielle J Smyth, Laura Cook, Steven F Ziegler, Megan K Levings, Rick M Maizels

**Affiliations:** ^1^ Wellcome Centre for Integrative Parasitology Institute of Infection, Immunity and Inflammation University of Glasgow Glasgow UK; ^2^ Department of Medicine BC Children’s Hospital Research Institute University of British Columbia Vancouver BC Canada; ^3^ Department of Translational Research Benaroya Research Institute Seattle WA USA; ^4^ Present address: Division of Cell Signalling and Immunology School of Life Science University of Dundee Dundee DD1 4HN UK; ^5^ Present address: The Peter Doherty Institute for Infection and Immunity University of Melbourne Melbourne VIC Australia

**Keywords:** autoimmunity, immune evasion, parasite cytokines, regulatory T cells

## Abstract

Transforming growth factor‐beta (TGF‐β) family proteins mediate many vital biological functions in growth, development and regulation of the immune system. TGF‐β itself controls immune homeostasis and inflammation, including conversion of naïve CD4^+^ T cells into Foxp3^+^ regulatory T cells (Tregs) in the presence of interleukin‐2 and T‐cell receptor ligands. The helminth parasite *Heligmosomoides polygyrus* exploits this pathway through a structurally novel TGF‐β mimic (*Hp*‐TGM), which binds to mammalian TGF‐β receptors and induces Tregs. Here, we performed detailed comparisons of *Hp*‐TGM with mammalian TGF‐β. Compared with TGF‐β, *Hp‐*TGM induced greater numbers of Foxp3^+^ Tregs (iTregs), with more intense Foxp3 expression. Both ligands upregulated Treg functional markers CD73, CD103 and programmed death‐ligand 1, but *Hp‐*TGM induced significantly higher CD39 expression than did TGF‐β. Interestingly, in contrast to canonical TGF‐β signaling through Smad2/3, *Hp‐*TGM stimulation was slower and more sustained. Gene expression profiles induced by TGF‐β and *Hp‐*TGM were remarkably similar, and both types of iTregs suppressed T‐cell responses *in vitro* and experimental autoimmune encephalomyelitis‐driven inflammation *in vivo*. *In vitro,* both types of iTregs were equally stable under inflammatory conditions, but *Hp*‐TGM‐induced iTregs were more stable *in vivo* during dextran sodium sulfate‐induced colitis, with greater retention of Foxp3 expression and lower conversion to a ROR‐γt^+^ phenotype. Altogether, results from this study suggest that the parasite cytokine mimic, *Hp‐*TGM, may deliver a qualitatively different signal to CD4^+^ T cells with downstream consequences for the long‐term stability of iTregs. These data highlight the potential of *Hp‐*TGM as a new modulator of T‐cell responses *in vitro* and *in vivo*.

## Graphical abstract

This study establishes that a helminth parasite protein fully mimics the immune‐suppressive effects of the mammalian cytokine transforming growth factor‐beta (TGF‐β). Named TGM (TGF‐β mimic), the parasite product drives Smad phosphorylation and induces suppressive regulatory T cells that are effective both *in vitro* and *in vivo*. Gene expression analysis confirms that despite the lack of structural similarity between TGF‐β and TGM, both ligands induce parallel responses in murine T cells. RA = Retinoic Acid.

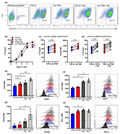

## INTRODUCTION

The family of transforming growth factor‐beta (TGF‐β) proteins constitute a widely expressed multifaceted set of mediators, which are essential for critical biological functions such as embryonic development, tissue repair and immune regulation.^1^, ^2^, ^3^ Virtually all cells express one or more members of the TGF‐β superfamily, as well as heterodimeric receptors specific for individual ligands. Fibroblasts, platelets, epithelial cells and T cells are, among others, notable producers of the cytokine TGF‐β; however, it is expressed as a latent inactive precursor, which is activated through tightly regulated proteolytic cleavage.^4^, ^5^ Moreover, activation of TGF‐β requires key membrane‐bound partners such as αvβ8 integrins and glycoprotein‐A repetitions predominant (GARP) that spatially constrain the immunosuppressive cytokine when interacting with receptors on target cells.^6^, ^7^, ^8^

TGF‐β signals through a heterodimeric complex composed of type I and II transmembrane serine/threonine kinase receptors (TβRI and II). Canonical signaling for TGF‐β occurs when active TGF‐β binds TβRII which then recruits and phosphorylates TβRI, resulting in downstream phosphorylation of cytosolic Smad2/3 complexes.^9^ Once Smad2/3 is phosphorylated, binding of Smad4 to the complex occurs, resulting in nuclear translocation and promoter activation to induce transcription of TGF‐β‐specific genes, the variety of which are dependent on the cell type and environment.^2^ Smad7, which acts as a negative regulator of the Smad2/3 pathway, is one of the few genes induced by TGF‐β signaling in all cell types.^10^

TGF‐β has a variety of immunological functions; from the view of immunoregulation, the role of TGF‐β in T‐cell differentiation is of particular interest. *In vitro,* TGF‐β signaling can promote CD4^+^ T‐cell polarization toward T helper (Th) 9 and Th17 phenotypes in the presence of interleukin (IL)‐4 and IL‐6, respectively.^11^, ^12^ Perhaps more notably, TGF‐β signaling is also essential for the induction of regulatory T cells (Tregs) in both the periphery and the thymus.^13^, ^14^, ^15^ *In vitro,* mammalian Tregs can be induced from naïve CD4^+^ T cells by T‐cell receptor/costimulatory engagement in the presence of IL‐2 and TGF‐β, and these cells are identifiable by the expression of their master transcription factor (Foxp3) and constitutive expression of high levels of CD25 (IL‐2Rα).^16^, ^17^

Tregs are critically important for regulation of the immune system.^18^, ^19^ In patients with inflammatory or autoimmune disorders, such as multiple sclerosis and rheumatoid arthritis, the frequency or function of Tregs may be reduced compared with healthy controls.^20^, ^21^ Therefore, innovative therapies now aim to promote Tregs, by either expanding their numbers *ex vivo* for infusion or directly *in vivo,* to suppress disease symptoms.^22^, ^23^ There are several hurdles that need to be overcome to successfully develop Treg therapies. Patients with inflammatory diseases may have reduced, or intrinsically dysfunctional, Treg populations from which to expand. Strategies promoting *in vivo* expansion also need to account for effects of an inflammatory environment as in the presence of IL‐6, Tregs can convert to a Th17 phenotype because of high expression of the IL‐6 receptor or STAT3‐dependent loss of Foxp3 expression, which could be problematic in autoimmune diseases such as multiple sclerosis, where inflammatory Th17 cells drive disease.^24^, ^25^ One method to overcome this would be to induce Tregs *ex vivo* from naïve CD4^+^ T cells using TGF‐β, for autologous cell therapy,^26^ which has an advantage, as TGF‐β treatment is thought to downregulate IL‐6R on induced Tregs (iTregs)^27^; in addition, it has been suggested that inclusion of retinoic acid (RA) *in vitro* iTreg cultures may render cells more resistant to effector cell conversion.^28^

Helminth parasites are known to induce Tregs during infection to potentially enhance their survival within the host, and one mechanism by which they do so is through the TGF‐β pathway.^29^, ^30^, ^31^ In particular, the murine intestinal nematode *Heligmosomoides polygyrus* secretes a protein, *Hp‐*TGM (TGF‐β mimic), which mimics the activity of TGF‐β and binds to its receptors despite having no structural homology.^32^ This novel parasite protein is an attractive potential therapeutic given that, unlike mammalian TGF‐β which is tightly regulated, *Hp‐*TGM is readily synthesized as an active protein and may be a more stable stimulator of TGF‐β pathways within the host. Furthermore, *Hp‐*TGM is one of 10 family members of proteins produced by *H*. *polygyrus* with similar gene sequences, indicating that activation of TGF‐β pathways is a positively selected mechanism to enhance parasite survival.^33^

In our previous studies, we identified that *Hp‐*TGM stimulates canonical TGF‐β signaling through pSmad2/3 and induces expression of Foxp3 in both mouse and human T cells.^32^ Here we used mouse T cells and compared the signaling profile, gene expression and stability of TGF‐β‐ and *Hp‐*TGM‐induced iTregs. We established that *Hp‐*TGM can induce robust iTregs to an equal or greater degree than TGF‐β, with a superior stability profile under inflammatory conditions. Parallel studies now being reported on human T cells demonstrate the induction of functionally suppressive iTregs by *Hp*‐TGM which showed greater *in vitro* stability than comparable cells induced by ΤGF‐β.^34^ As *Hp*‐TGM is highly stable, does not require bioactivation and may not be subject to the same constraints as TGF‐β, it has the potential to offer a novel therapeutic to manipulate T‐cell responses in human disease.

## RESULTS

### *Hp‐*TGM potently induces murine Foxp3^+^ Tregs *in vitro*

**Figure 1:**
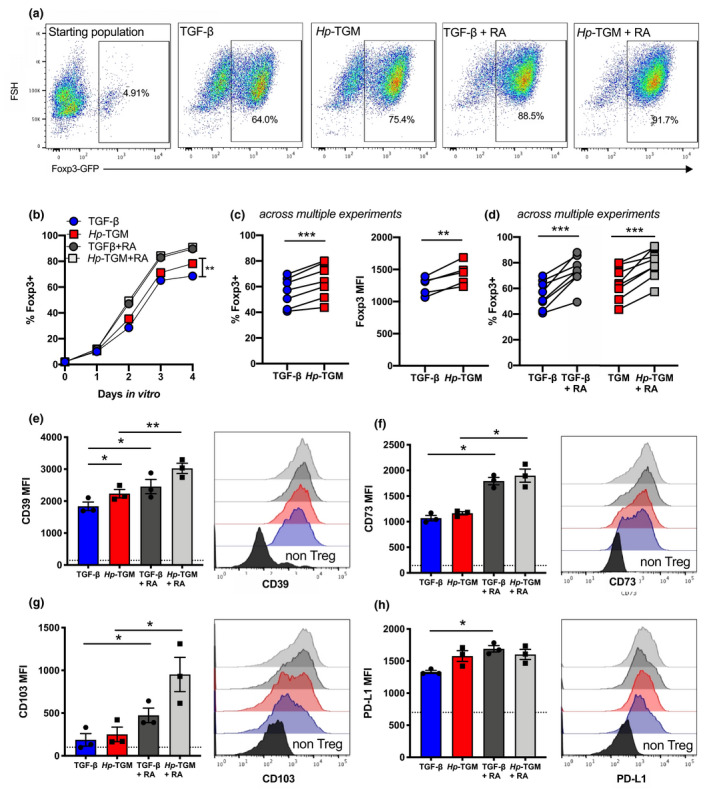
*Hp‐*TGM and TGF‐β induce Foxp3^+^ iTregs with similar phenotypes. CD4^+^ T cells were isolated from the spleens of naïve male Foxp3‐GFP reporter BALB/c mice and cultured in the presence of plate‐bound anti‐CD3, IL‐2 with or without RA and either *Hp‐*TGM or TGF‐β for up to 4 days. **(a)** Representative expression of Foxp3‐GFP in the starting population (far left panel), and at day 4 for each treatment group, pregated on lymphocytes, single cells, live cells and CD3^+^CD4^+^ T cells. **(b)** Percentage of Foxp3(GFP)^+^ Tregs induced *in vitro* over time. **(c, d**) Percentage of induced Foxp3^+^ Tregs and Foxp3 MFI at day 4; data shown are from multiple experiments **(c)** without RA or **(d)** with RA addition. **(e–h)** After 4 days of culture the MFI of **(e)** CD39, **(f)** CD73, **(g)** CD103 and **(h)** PD‐L1 was assessed on Foxp3^+^ Tregs. Expression levels on the CD4^+^ T‐cell starting population are shown as a dashed horizontal line in bar charts and in solid black on histograms. Data shown are the mean of three biological replicates per sample from 3 or 5 independent experiments; error bars represent mean ± s.e.m. A paired *t*‐test was used to compare each set of groups. *P < 0.05, **P < 0.01, ***P < 0.001. GFP, green fluorescent protein; *Hp*‐TGM, TGF‐β mimic; iTregs, induced Tregs; MFI, mean fluorescence intensity; PD‐L1, programmed death‐ligand 1; RA, retinoic acid; TGF‐β, transforming growth factor‐beta; Tregs, regulatory T cells.

To compare the efficacy of *Hp‐*TGM and TGF‐β for *in vitro* Treg induction, CD4^+^Foxp3^–^ T cells were sorted from Foxp3‐green fluorescent protein (GFP) reporter mice^35^ and cocultured with IL‐2, anti‐CD3 and either TGF‐β or *Hp‐*TGM. Aliquots of these cells were collected every 24 h and evaluated for Foxp3 induction using the gating strategy shown in Figure 1a. We found that both proteins induced progressively more Foxp3^+^ cells over time, with the effect of *Hp*‐TGM significantly greater than TGF‐β by day 4 (Figure 1b) in repeated comparisons (Figure 1c). The degree of Foxp3 expression in iTregs showed a similar trend between *Hp‐*TGM and TGF‐β, with *Hp‐*TGM iTregs expressing higher levels, as determined by mean fluorescence intensity across multiple comparisons (Figure 1c). Parallel cultures were also grown in the presence of RA, a known enhancer of Treg induction^36^; we found that RA maximized Foxp3 expression and equalized the effects of TGF‐β compared with *Hp*‐TGM (Figure 1b, d).

In addition, expression levels of Treg‐associated functional markers CD39, CD73, CD103 and programmed death‐ligand 1 were measured, showing *Hp‐*TGM and TGF‐β had very similar effects on increasing expression of these molecules, with the adenosine triphosphatase/adenosine diphosphatase ectoenzyme CD39 having significantly higher expression on *Hp‐*TGM iTregs, compared with TGF‐β iTregs (as assessed by a paired comparison; Figure 1e). The presence of RA significantly enhanced the expression of all of these markers in both TGF‐β and *Hp‐*TGM cultures (Figure 1e–h), indicating that the addition of RA not only induces more iTregs but also increases expression of markers associated with suppressive function.

### *Hp‐*TGM induces sustained canonical signaling through TGF‐β receptors

**Figure 2:**
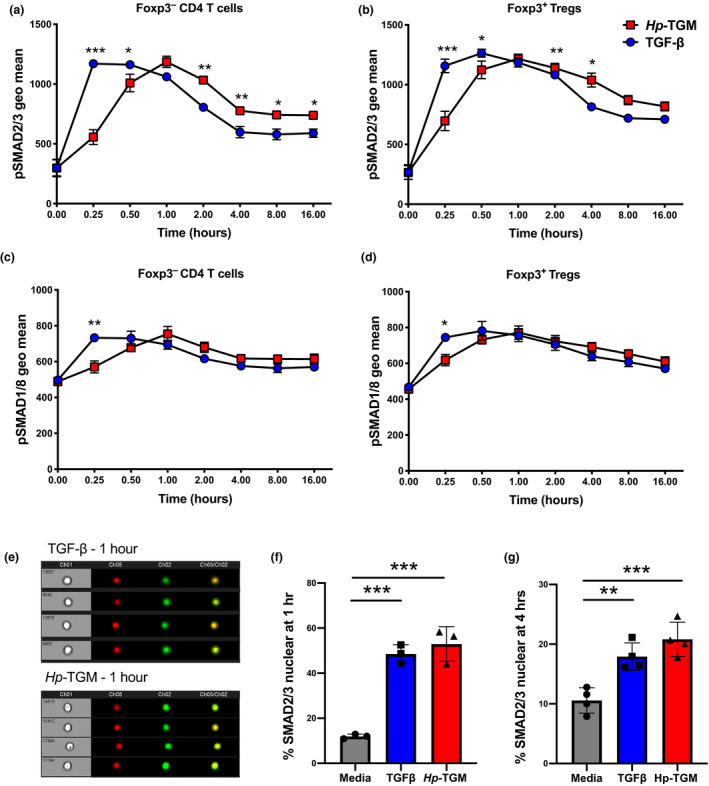
Activation of pSMAD2/3 and pSMAD1/8 signaling pathways by *Hp‐*TGM and TGF‐β. CD4^+^ T cells were isolated from the spleens of female C57BL/6 mice and cultured in serum‐free media for 4 h prior to the addition of *Hp‐*TGM or TGF‐β, for 15 min to 16 h. Expression of pSMAD2/3 and pSMAD1/8 was determined by flow cytometry using barcode‐labeled beads as shown in Supplementary figure 1. **(a)** pSMAD2/3 expression in non‐Treg Foxp3^–^ CD4^+^ T cells. **(b)** pSMAD2/3 expression in Foxp3^+^ Tregs, **(c)** pSMAD1/8 expression in Foxp3^‐^ CD4^+^ T cells and **(d)** pSMAD1/8 expression in Foxp3^+^ Tregs. **(e)** Raw ImageStream data showing SMAD2/3 nuclear localization during TGF‐β receptor activation showing brightfield, nuclear dye DRAQ5 in red and Smad2/3‐AF488 in green. Cells that are SMAD2/3 nuclear located have overlapping signal for both fluorochromes. **(f, g)** Percentage of CD4^+^ T cells that had SMAD2/3 nuclear localized at **(f)** 1 h and **(g)** 4 h post‐*Hp‐*TGM or TGF‐β stimulation. Data shown are representative of three independent experiments, with three biological replicates per timepoint (*n* = 3, mean ± s.e.m.); **a–d** were tested using a two‐way ANOVA with Šidák’s multiple comparisons test. **f** and **g** were tested using a one‐way ANOVA with Tukey’s multiple comparisons test; *P < 0.05, **P < 0.01, ***P < 0.001. *Hp*‐TGM, TGF‐β mimic; TGF‐β, transforming growth factor‐beta; Tregs, regulatory T cells.

*Hp*‐TGM binds mammalian TGF‐β receptors and stimulates a qualitatively similar level of intracellular Smad2/3 phosphorylation, as judged by western blotting and flow cytometry at 16–18 h poststimulation.^32^ To study signaling events in more detail, we used phospho‐Smad2/3‐specific antibodies in flow cytometry at doubling intervals from 15 min following addition of either *Hp*‐TGM or TGF‐β to freshly isolated mouse CD4^+^ T cells (example gating strategy is shown in Supplementary figure 1) and assessed pSmad2/3 in Foxp3^–^ (Figure 2a) and Foxp3^+^ (Figure 2b) subsets. In both cell subsets, TGF‐β elicited a much more rapid response, peaking at 15–30 min and slowly declining thereafter, whereas *Hp*‐TGM drove a slower but more sustained response, which in CD4^+^Foxp3^–^ T cells was significantly higher throughout the period 2–16 h poststimulation (Figure 2a).

Signaling through TGF‐β receptors can also occur through other noncanonical pathways, including via Smad1/5/8 phosphorylation.^37^ Flow cytometry analysis of T cells over the same time course again showed more rapid activation of pSmad1/8 by TGF‐β compared with *Hp*‐TGM (Figure 2c, d), although at all time points from 30 min onward, Smad1/8 phosphorylation was similar with both ligands.

Phosphorylated Smad2/3 forms a complex with the common Smad, Smad4, which then moves into the nucleus to directly regulate gene transcription and expression of target genes.^38^ Therefore, we also evaluated Smad signaling by imaging flow cytometry to determine the relative level of cytoplasmic and nuclear localization of Smad2/3 proteins after freshly isolated mouse CD4^+^ T cells were stimulated with *Hp‐*TGM or TGF‐β (gating strategy examples are presented in Supplementary figure 2, Figure 2e). At both 1 and 4 h poststimulation, total CD4^+^ T cells showed a greatly enhanced degree of nuclear Smad2/3 translocation with both treatments (Figure 2f, g).

### *Hp‐*TGM and TGF‐β induce similar expression of Treg‐related genes

**Figure 3:**
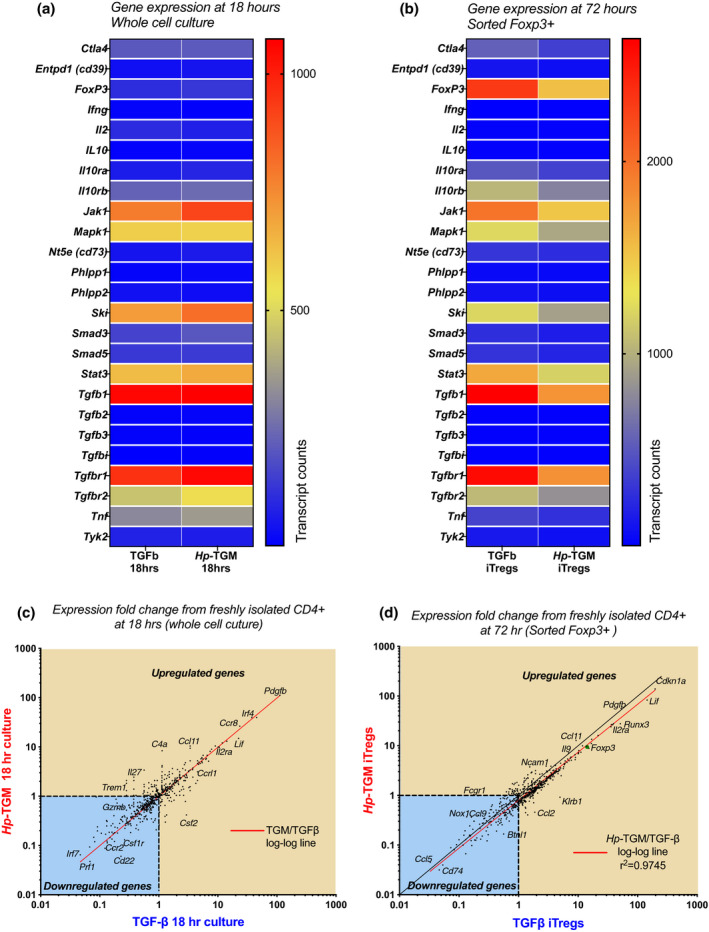
*Hp‐*TGM‐ and TGF‐β‐induced Foxp3^+^ Tregs had similar gene expression after 3 days in culture. CD4^+^ T cells were isolated from the spleens of naïve Foxp3‐GFP reporter BALB/c mice and cultured in the presence of anti‐CD3/anti‐CD28 beads, IL‐2 and either *Hp‐*TGM or TGF‐β for 18 or 72 h. Gene expression was analyzed using the mouse immunology gene set (V1) from NanoString on total cell populations at 18 h, and on cells sorted for Foxp3‐GFP expression at 72 h. **(a)** Heatmap showing the gene expression of isolated CD4^+^ T cells that were cultured in the presence of *Hp‐*TGM or TGF‐β for 18 h prior to analysis, with the blue color indicating a low level of gene expression and red color indicating a high level of gene expression as determined by the number of transcript counts. **(b)** Heatmap showing the gene expression of TGF‐β‐induced Tregs (TGF‐β iTregs) and *Hp‐*TGM‐induced Tregs (*Hp‐*TGM iTregs) which were sorted for Foxp3‐GFP expression after 72 h of culture prior to gene analysis. **(c, d)** Fold‐change comparison of genes from 18‐h‐cultured CD4^+^ T cells (**c**) and 72‐h Foxp3^+^‐sorted T cells **(d)**, cocultured with *Hp‐*TGM or TGF‐β, relative to the freshly sorted CD4^+^ T‐cell starting population. The log–log line is indicated in red, and top right quadrant shows genes that are upregulated compared with fresh CD4^+^ control cells, and the blue‐shaded lower left quadrant indicates those that are downregulated. GFP, green fluorescent protein; *Hp*‐TGM, TGF‐β mimic; IL, interleukin; iTregs, induced Tregs; TGF‐β, transforming growth factor‐beta; Tregs, regulatory T cells.

TGF‐β activation of T cells results in a complex web of gene expression changes beyond the Smad‐dependent pathway.^5^ To ascertain whether *Hp*‐TGM replicated, or altered, these transcriptional profiles we conducted NanoString digital gene expression analysis on unstimulated *ex vivo* CD4^+^ non‐Tregs (Foxp3^–^) and Foxp3^+^ Tregs, as well as Foxp3^+^ Tregs induced *in vitro* with either *Hp*‐TGM or TGF‐β. First, we focused on a panel of genes associated with Treg function and assessed the expression of these genes after *ex vivo* isolated Foxp3^–^ cells were cultured for 18 or 72 h in the presence of either *Hp*‐TGM or TGF‐β. At 18 h, prior to the induction of *Foxp3*, total cell cultures were analyzed, revealing an upregulation of *Jak1,* *Mapk1, Ski, Tgfb1, Tgfb2* and to a lesser extent *Tnf*, genes to similar levels in both TGF‐β‐ and *Hp*‐TGM‐treated cells (Figure 3a). After 72 h of culture, we sorted cells according to their Foxp3‐GFP status and separately analyzed their gene expression.

Within the Foxp3^+^ sorted cells, we found similar upregulation of genes associated with Treg function by both TGF‐β and *Hp‐*TGM iTregs, including levels of *Foxp3* (Figure 3b). We then evaluated gene expression as a fold change compared with freshly isolated CD4^+^ T cells, and found that cells cocultured with TGF‐β or *Hp*‐TGM, both total T cells at 18 h, and fluorescence‐activated cell sorting (FACS)‐sorted Foxp3^+^ iTregs at 72 h, showed a close correlation in nearly all genes evaluated as indicated by the red line (Figure 3c, d).

From cultures stimulated with TGF‐β or *Hp*‐TGM we also isolated the Foxp3^–^ cells to identify any differences underlying fate decisions with *Hp‐*TGM. In these populations we found that both ligands amplified the same gene set, with the exception of *Foxp3* (Supplementary figure 3). We noted inverse regulation of *Jak1, Ski* and *Tgfbr1*, which were more strongly expressed in TGF‐β iTregs and in *Hp*‐TGM‐stimulated CD4^+^Foxp3^–^ cells (Figure 3b, Supplementary figure 4). Given that Ski protein is a negative suppressor of TGF‐β signaling through the repression of Smad4,^39^ these results suggest that the *Ski* inhibition pathway may be differentially activated by TGF‐β, or that *Ski*‐expressing naïve T cells are more resistant to Foxp3 induction by *Hp‐*TGM, possibilities that require further investigation. Taken as a whole, however, these results demonstrate that the gene pathways induced by *Hp‐*TGM and TGF‐β are highly similar, confirming that the parasite mimic effectively induces a parallel suppressive gene program.

### Both *Hp‐*TGM‐ and TGF‐β‐induced Foxp3^+^ Tregs can suppress Τ‐cell responses *in vitro* and *in vivo*

**Figure 4:**
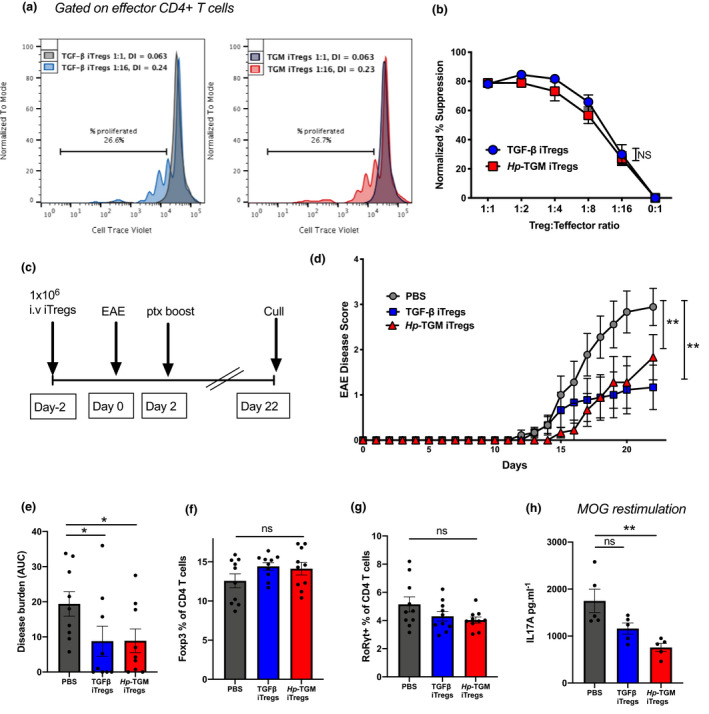
*Hp‐*TGM‐ and TGF‐β‐induced Foxp3^+^ Tregs were equally able to suppress T‐cell responses *in vitro* and reduce EAE severity *in vivo*. Naïve CD4^+^ T cells from **(a, b)** Foxp3‐GFP C57BL/6 or **(c, d)** IL‐10‐GFP‐Foxp3‐RFP C57BL/6 mice were cultured to generate *Hp‐*TGM‐ or TGF‐β‐induced Foxp3^+^ Tregs (iTregs) that were sorted based on Foxp3‐GFP/RFP expression and used for downstream analysis *in vitro* and *in vivo*. **(a)** Suppression assays were performed using freshly isolated and Cell Trace Violet‐labeled responder CD4^+^ T cells cultured with anti‐CD3/CD28 beads and varying concentrations of *Hp‐*TGM or TGF‐β iTregs. The percentage of T cells that proliferated in the presence of iTregs at a ratio of 2:1 (iTregs to labeled CD4^+^ T cells (gray)) and either 1:16 ratio of TGF‐β Tregs (blue) or TGM iTregs (red) where the percentage proliferation is shown as well as the division index (DI) calculated using FlowJo analysis software v9 (BD Biosciences). **(b)** Percentage suppression of effector cell proliferation is shown for varying ratios of iTregs added to cultures, relative to proliferation in cultures of effector cells alone. **(c)** Female C57BL/6 mice received 1 × 10^6^ of either *Hp‐*TGM or TGF‐β iTregs or PBS on day −2 and then were immunized for EAE on days 0 and 2, and euthanized at day 22. **(d)** Disease scores from mice receiving iTregs showed a marked decrease in severity by day 16 post‐EAE induction, which is further indicated by the overall disease burden **(e)**. The percentage of **(f)** Foxp3^+^ Tregs and **(g)** RORγt^+^ CD4^+^ T cells in the inguinal lymph node. **(h)** Splenocytes that were restimulated for 72 h in the presence of the immunizing antigen (MOG) showed a significant reduction in IL‐17A production in mice that were treated with *Hp‐*TGM iTregs. Data are from one of two similar independent experiments (**a, b, h**: *n* = 3 or 5 per experiment group) or pooled from 2 independent experiments (**c–g**: *n* = 9 or 10 per treatment group). Data were analyzed by two‐way ANOVA with Dunnett’s multiple comparisons test comparing the iTreg treatment groups with the control PBS group (**d**), *P < 0.05, **P < 0.01. EAE, experimental autoimmune encephalomyelitis; GFP, green fluorescent protein; *Hp*‐TGM, TGF‐β mimic; IL, interleukin; iTregs, induced Tregs; NS, not significant; PBS, phosphate‐buffered saline; RFP, red fluorescent protein; TGF‐β, transforming growth factor‐beta; Tregs, regulatory T cells.

We next tested the ability of iTregs induced by *Hp*‐TGM to suppress immune responses *in vitro* and *in vivo*. *In vitro*, both *Hp*‐TGM‐ and TGF‐β‐induced Tregs very effectively reduced the proliferation of cocultured CD4^+^ T cells activated by anti‐CD3/anti‐CD28 beads in a dose‐dependent manner (Figure 4a, b). To assess the ability of iTregs to suppress T‐cell responses *in vivo*, we used a T‐cell model of autoimmunity, experimental autoimmune encephalomyelitis (EAE), in which Th17 CD4^+^ cells play a critical role in driving disease pathology through IL‐17 secretion.^40^, ^41^ Mice are primed to myelin oligodendrocyte glycoprotein (MOG) peptide and develop ascending paralysis.^42^ Two days prior to priming, animals received 1 × 10^6^ *Hp*‐TGM‐ or TGF‐β‐induced Tregs, and the course of EAE disease was followed for 22 days (Figure 4c). Symptoms first began to appear from day 13, and although *Hp*‐TGM‐ and TGF‐β‐induced Tregs did not fully abrogate pathology, both ameliorated EAE disease severity (Figure 4d, e). There was no significant enhancement of overall Treg numbers or reduction of the percentage of CD4^+^ T cells expressing the Th17‐associated transcription factor RORγt^+^ in either iTreg‐treated group (Figure 4f, g). There was, however, a significant reduction in the amount of IL‐17A secreted by cultured cells from *Hp‐*TGM iTreg‐treated mice following *ex vivo* MOG restimulation (Figure 4). These results indicate that while both types of iTregs are suppressive *in vitro* and *in vivo,* the *Hp‐*TGM iTregs may be better able to suppress cytokine secretion by inflammatory cells.

### *Hp‐*TGM‐ and TGF‐β‐induced Foxp3^+^ Tregs display similar levels of stability *in vitro*

**Figure 5:**
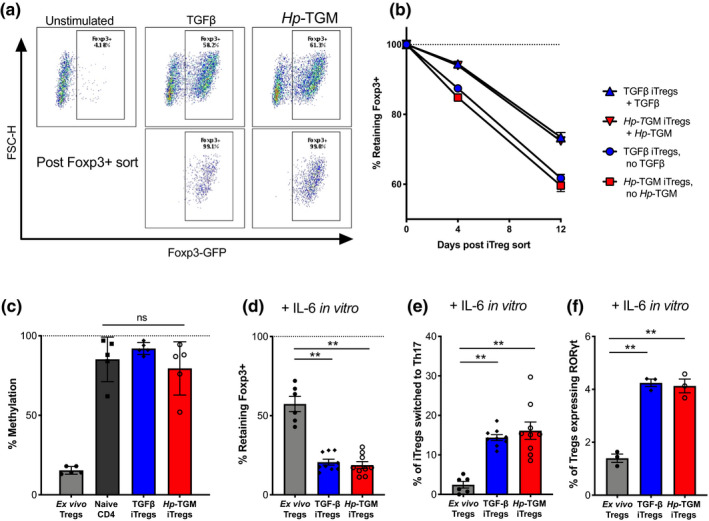
*Hp‐*TGM‐ and TGF‐β‐induced Foxp3^+^ Tregs showed similar levels of methylation and stability, and acted similarly under inflammatory conditions *in vitro*. **(a)** Naïve CD4^+^ T cells from male BALB/c‐Foxp3‐GFP mice were cultured with *Hp‐*TGM or TGF‐β to induce Foxp3^+^ Tregs (iTregs) that were sorted based on Foxp3‐GFP expression and used for downstream analysis *in vitro* as shown by the representative flow cytometry plots. **(b)** Percentage retention of Foxp3 expression among sorted Foxp3 loss over time in culture for *Hp‐*TGM‐ and TGF‐β‐induced Tregs in the presence of additional IL‐2, with or without *Hp‐*TGM/TGF‐β in the second step culture. **(c)** *In vivo* differentiated Foxp3^+^ Tregs (nTregs) show a high level of demethylation at the TSDR locus; however, *Hp‐*TGM‐ and TGF‐β‐induced Tregs after 12 days in culture have a similar level of methylation in freshly isolated naïve CD4^+^ T cells. **(d)** Percentage of IL‐6‐induced Foxp3 loss in sorted *Hp‐*TGM or TGF‐β‐iTregs that were cultured in the presence of IL‐6 for 4 days. **(e)** Percentage of iTreg cultures in **d** that converted to Th17 as evaluated by their expression of both RORγt and IL‐17A in the presence of IL‐6. **(f)** Percentage of iTregs that became double positive for RORγt and Foxp3 in the presence of IL‐6 for 4 days. Data are from three similar independent experiments (**a–f**). Shown are the means ± s.e.m. and data were analyzed using a one‐way ANOVA (**c–f**) with Dunnett’s multiple comparisons test comparing iTreg treatment groups with nTreg controls, and a paired *t*‐test between TGF‐β and TGF‐β + RA. **P < 0.01. FSC‐H, forward scatter‐height; GFP, green fluorescent protein; *Hp*‐TGM, TGF‐β mimic; IL, interleukin; iTregs, induced Tregs; NS, not significant; TGF‐β, transforming growth factor‐beta; Th, T helper; Tregs, regulatory T cells; TSDR, Treg‐specific demethylation region.

Although Foxp3 expression can readily be induced in murine T cells through the TGF‐β signaling pathway, in the absence of continued TGF‐β activation, a portion of cells reverts to the Foxp3‐negative state.^43^ To assess the ability of iTregs to retain their Foxp3 expression over time, TGF‐β and *Hp‐*TGM iTregs were induced from CD4^+^ T cells, using the previously defined protocol (Figure 1), and then sorted based on Foxp3‐GFP expression to achieve a population that was over 99% Foxp3^+^ cells (Figure 5a). These cells were then cultured for up to 12 days in the presence of high levels of IL‐2 (1000 U/mL) with, or without, additional TGF‐β or *Hp‐*TGM, after which time both iTreg groups lost similar levels of Foxp3 expression with a much greater decline seen in the groups without additional cytokines added (Figure 5b).

As epigenetic changes are strongly associated with Treg stability,^44^ we investigated the degree of methylation at the Foxp3 locus, which is reduced in stable (*in vivo* differentiated) Tregs (Figure 5c). We measured methylation levels at the Treg‐specific demethylation region (TSDR) in the first intron of the Foxp3 locus in iTregs at 12 days postinduction, but neither TGF‐β nor *Hp*‐TGM drove any significant demethylation (Figure 5c), indicating that under the conditions used to induce functional murine iTregs, we do not observe the demethylation seen in established *in vivo* populations of Tregs.

Foxp3 expression is reported to be particularly fragile in inflammatory environments,^45^ so we next tested its stability *in vitro* in the presence of IL‐6. There was a substantial loss of Foxp3 expression in both TGF‐β‐ and *Hp*‐TGM‐induced iTregs (approximately 80% loss, Figure 5d), with approximately 15% of both iTregs converting to a Th17 cell phenotype (Figure 5e), classified as Foxp3^–^ cells that expressed both the transcription factor RORγt and secreted IL‐17A. Furthermore, freshly isolated *ex vivo* CD4^+^CD25^+^Foxp3^+^ Tregs with low levels of TSDR methylation were less susceptible to Foxp3 loss and adoption of a Th17 cell phenotype in the presence of IL‐6, indicating that TGF‐β/*Hp*‐TGM‐induced iTregs have reduced Treg‐lineage stability in the presence of IL‐6 (Figure 5c). Interestingly, IL‐6 stimulation of both TGF‐β and *Hp*‐TGM iTregs induced a small population of Foxp3^+^RORγt^+^ cells, more so than freshly isolated Tregs (Figure 5f). This dual positive population has been shown to be a stable Treg lineage with a high suppressive capacity, particularly during the inflammation of mucosal sites such as in the T‐cell transfer colitis model where transfer of Foxp3^+^RORγt^+^ Tregs show a more potent suppression compared with Foxp3^+^RORγt^‐^ Tregs.^46^

### *Hp‐*TGM‐ and TGF‐β‐induced Foxp3^+^ Tregs display similar levels of stability *in vivo*

**Figure 6:**
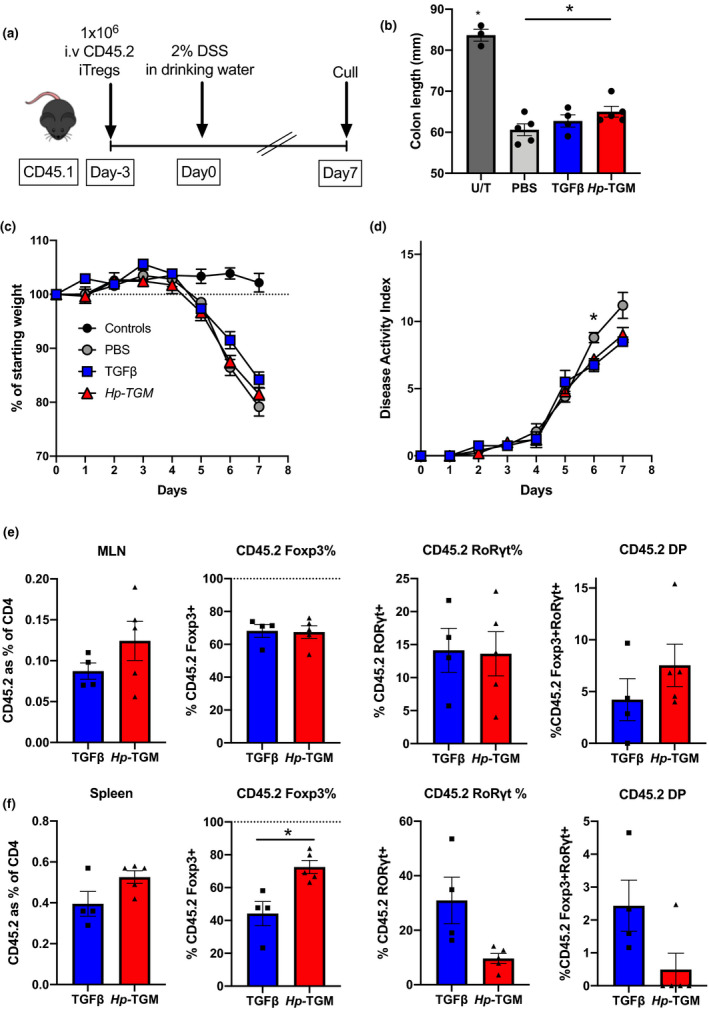
*Hp‐*TGM‐ and TGFβ‐induced Tregs showed similar levels of stability *in vivo* using DSS‐induced colitis as an inflammatory model. Male CD45.1 congenic (Ly5.1) mice received either *Hp‐*TGM‐ or TGF‐β‐induced Tregs from Foxp3‐GFP C57BL/6 mice or PBS only 3 days prior to DSS colitis induction. Colitis was induced by administering 2% DSS in the drinking water for 7 days and the mice were euthanized and transferred Tregs were analyzed. **(a)** The DSS colitis experimental protocol where *Hp‐*TGM or TGF‐β iTregs from male C57BL/6‐Foxp3‐GFP mice were induced *in vitro* and sorted for Foxp3 expression prior to intravenous injection into male CD45.1 mice 3 days prior to DSS administration. **(b)** Colon length of DSS colitis mice compared with untreated (U/T) control mice. **(c)** Percentage of initial body weight of colitis mice over time. **(d)** Colitis disease severity score [disease activity index (DAI)] over time. **(e)** Analysis of transferred iTregs (CD45.2‐expressing cells) in the mesenteric lymph node (MLN), indicating percentage of CD45‐2 among all CD4^+^ T cells, percentage of cells which retained Foxp3 expression, percentage of cells that turned on RORγt and the percentage of cells which became double positive (DP) for both Foxp3 and RORγt. **(f)** Analysis of transferred cells in the spleen, as above. Data are from one or two similar independent experiments (*n* = 2 or 4 per experiment group). A one‐way ANOVA **(c)** with Bonferroni’s multiple comparisons test, a two‐way ANOVA **(d)** with Tukey’s multiple comparisons test and an unpaired *t*‐test **(e, f)**. *P < 0.05. DSS, dextran sodium sulfate; GFP, green fluorescent protein; *Hp*‐TGM, TGF‐β mimic; iTregs, induced Tregs; PBS, phosphate‐buffered saline; TGF‐β, transforming growth factor‐beta; Tregs, regulatory T cells.

We then evaluated iTreg stability *in vivo*, again within an inflammatory environment using an acute model of colitis induced by a mixture of dextran sodium sulfate (DSS) in drinking water (Figure 6a). In this model, mice begin to lose weight within 5 days, and the degree of pathology is measured over time as a disease activity index. Donor TGF‐β or *Hp‐*TGM‐induced Tregs, carrying the allotypic marker CD45.2, were transferred into CD45.1 recipient mice 3 days before commencement of DSS treatment to allow the iTregs to engraft prior to induced disease. Recipient mice showed little protection against colon shortening (Figure 6b), weight loss (Figure 6c) or disease score as measured by a disease activity index (Figure 6d), which is unsurprising given that DSS‐induced colitis is considered to be triggered initially by innate inflammation, although other studies have noted some reduction in DSS‐induced colitis with Treg treatment.^47^, ^48^

Importantly, this model allowed us to assess the stability of donor iTregs by immunophenotyping after 7 days of DSS treatment *in vivo*. Cells were isolated from the mesenteric lymph nodes and spleens of all recipient mice, with no significant differences in numbers (Supplementary figure 5). Lymph node populations showed similar levels of Foxp3 retention or conversion to a Th17 phenotype and although not statistically significant, levels of Foxp3^+^RORγt^+^ double‐positive cells were increased following transfer of *Hp‐*TGM‐induced Tregs compared with TGF‐β‐induced Tregs (Figure 6e). However, within the systemic response as measured by transferred cells recovered from the spleen, there was significantly greater stability of Foxp3 expression, and a trend toward reduced RORγt expression, on *Hp‐*TGM iTregs compared with TGF‐β‐induced Tregs (Figure 6f). Altogether, our results establish that the parasite cytokine *Hp‐*TGM is able to induce a Treg population that is equal to, and in some respects more stable than, TGF‐β‐induced Tregs *in vivo*, particularly under inflammatory conditions.

## DISCUSSION

TGF‐β is a pivotal cytokine in the immune system, restraining reactivity, dampening inflammation and restoring homeostasis through cross‐talk with multiple other growth factors.^1^, ^5^ Its essential role is evidenced not only by the lethality of gene deficiency in mutant mice,^49^, ^50^ but also by the high degree of conservation across the vertebrate subphylum.^50^ The potent inhibitory effects of TGF‐β, mediated in part through regulatory T cells, are also reflected in the elaborate control mechanisms which ensure that the processed ˜110‐amino acid mature TGF‐β is activated only under appropriate conditions.^7^ In this context, it is perhaps not surprising that pathogens manipulate the TGF‐β pathway, as described for example in malarial^51^ and filarial^52^ parasites, but few specific pathogen‐encoded mediators have yet been identified.

In the case of the murine intestinal helminth parasite *H. polygyrus*, we previously reported that the secretion of a ˜400‐amino acid protein composed of five domains, requiring no processing or activation, was able to ligate mammalian TGF‐β receptors and induce both murine and human Foxp3^+^ regulatory T cells *in vitro*.^32^ Remarkably, the parasite ligand lacked any structural or sequence similarity to the TGF‐β gene family, bearing a distant resemblance instead to complement control protein modules. This new parasite protein, named *Hp‐*TGM, is a monomer with direct binding capacity for both subunits of the mammalian signaling receptors (TβRI and TβRII). By contrast, the host ligand is a homodimeric protein which mediates an ordered assembly, binding first to TβRII, forming a complex which then recruits TβRI.^9^, ^53^, ^54^ In addition, mammalian TGFβ, but not *Hp‐*TGM, binds the TβRIII coreceptor betaglycan, enhancing activation through the signaling receptors.^55^, ^56^ Hence, it may be expected that *Hp*‐TGM‐induced activation may differ through altered receptor complex formation or stoichiometry, interactions with coreceptors and/or internalization of the bound receptors.

These structural and functional contrasts prompted us to conduct a more detailed comparison, reported here, of the ability of *Hp‐*TGM and TGF‐β to induce regulatory T cells *in vitro*. In every respect, from Foxp3 induction to altered gene expression and ability to suppress inflammatory responses, we find that *Hp*‐TGM is equal to, and in some cases more potent than, TGF‐β. Interestingly in view of the differing receptor interactions, signal induction by *Hp*‐TGM is relatively slow; this may reflect its higher affinity for TβRI that would still require interaction with TβRII for phosphorylation and subsequent activation of Smad intermediaries. Equally, the more sustained signaling with *Hp*‐TGM may explain the instances in which its effects appear more profound than those of TGF‐β, such as significantly higher Foxp3 expression in *Hp*‐TGM iTregs.^32^

In addition to the *in vitro* setting, in which *Hp‐*TGM fully replicates the functions of mammalian TGF‐β, we also tested the efficacy of iTregs induced by *Hp‐*TGM in *in vivo* models. In the mouse EAE model, disease was equally well suppressed by iTregs generated with either ligand, with a greater inhibition of autoantigen‐specific Th17 responses in mice receiving *Hp*‐TGM‐iTregs. Interestingly, in the context of EAE, suppression of IL‐17 production is restricted to the CD39^+^ subset of Foxp3^+^ cells,^57^ which we found to be more strongly upregulated by *Hp*‐TGM. Moreover, CD39 is an ectoenzyme that degrades extracellular adenosine triphosphate, thereby reducing proinflammatory purinergic signaling, while promoting Treg migration into the central nervous system.^58^ We also utilized the DSS model of colitis to create an inflammatory environment that challenges the stability of iTregs; in this setting *Hp*‐TGM iTregs did confer a small degree of protection and showed a significantly higher degree of retention of Foxp3 expression among splenic cells than observed in recipients of TGF‐β‐iTregs. We note that, in human Tregs, higher CD39 expression is associated with greater stability when exposed to the same inflammatory cytokines tested in our experiments.^59^

These studies address, but do not resolve, the question of how iTregs can best be generated that prove resilient in the face of inflammatory stimuli and would retain regulatory function after transfer to a host with inflammatory disorders.^23^, ^60^ Further modifications to Treg induction conditions are being explored, testing combinations of metabolic and epigenetic factors which are implicated in Treg commitment and function,^61^, ^62^, ^63^, ^64^ and varying the nature of the stimuli delivered *in vitro*. In the latter respect, it is interesting that when anti‐CD28 is omitted from the initial T‐cell cultures, thereby minimizing protein kinase C–NF‐κB signaling, newly induced iTregs do display the hypomethylation characteristic of more stable peripheral Tregs.^65^

It is important to note that our analysis thus far has focused on the effects in T cells, in order to replicate the regulatory pathways known to be induced by TGF‐β. It will be instructive to evaluate the effects of *Hp‐*TGM on a range of other cell types, in particular the gut epithelium as well as macrophages and other innate cells. The helminth protein is released during the adult life stage within the gut of the host, and this is where the highest concentrations of *Hp‐*TGM would be found, thus it would be of interest to assess whether the parasite mimic has a potent effect on these cell types in comparison to mammalian TGF‐β. Recent studies have also identified that TGF‐β can induce phosphorylation of the Smad1/5 pathway through two type I receptors, TGFBR1 and ACVR1 (one of the classical bone morphogenetic protein type I receptors), and activation through this pathway is required for full TGF‐β transcript activation.^66^ Our study showed that both TGF‐β and *Hp‐*TGM induced phosphorylation of Smad1/5/8; however, the signal was quite low, so it would be worth assessing the activation of this pathway in other cell types as well.

In conclusion, we describe a salient example of convergent evolution and cytokine mimicry, involving a strongly immunosuppressive protein at the center of immunological regulation. In this setting there is potential for its use as an anti‐inflammatory agent, particularly as parallel studies with human CD4^+^ T cells indicate efficient Foxp3 induction by *Hp*‐TGM even among memory effector T cells, with greater suppressive capacity and enhanced stability in comparison to TGF‐β iTregs.^34^ The ability of *Hp*‐TGM to induce human Tregs suggests that it may be therapeutically valuable, either directly *in vivo* or indirectly by *ex vivo* production of Tregs in an autologous transfer protocol,^23^, ^26^ and further investigations toward this goal are currently under way.

## METHODS

### Mouse strains and husbandry

Female and male Foxp3‐GFP C57BL/6, Foxp3‐GFP BALB/c, IL‐10‐GFP‐Foxp3‐red fluorescent protein (RFP) C57BL/6, CD45.1 C57BL/6 and C57BL/6 mice (aged 8–14 weeks) were used for experiments. All mice were either bred in‐house or sourced from the University of Edinburgh and housed in the animal facility at the University of Glasgow. All experiments were performed under UK Home Office license and approved by the University of Glasgow Ethical Review Board.

### *In vitro* Treg induction

A single‐cell suspension was prepared from the spleens of naïve mice by passing the tissue through a 70‐μm cell strainer; contaminating red blood cells were removed by resuspending the cells in red blood cell lysis buffer (Sigma, St Louis, MO, USA) for 2 min at room temperature. Cells were then washed and resuspended in media made up of Dulbecco’s modified Eagle’s medium containing HEPES, supplemented with 2 mM l‐glutamine, 10% heat‐inactivated fetal bovine serum (FBS), non‐essential amino acids, 100 U mL^−1^ of penicillin and 100 μg mL^−1^ of streptomycin (all purchased from Thermo Fisher Scientific, Waltham, MA, USA). The cell suspension was then enriched for CD4^+^ T cells using the autoMACS system (Miltenyi Biotec, Bergisch Gladbach, Germany) and the mouse CD4^+^ T cell isolation kit with “depletes” setting as per manufacturer’s instructions. Cells were cultured at a concentration of 2 × 10^5^ mL^−1^ in flat‐bottomed 96‐well plates (Corning, New York, NY, USA) coated with 10 μg mL^−1^ α‐CD3 (Life Technologies, Carlsbad, CA, USA) for flow analysis or 7.5 × 10^5^ cells in a 24‐well plate (Life Technologies) for cell sorting experiments. Additional IL‐2 (Miltenyi Biotec) was added at a final concentration of 400 U mL^−1^ and cytokines/proteins were added at 20 ng mL^−1^ for *Hp‐*TGM (expressed in HEK293 cells as previously described^32^) and hTGFβ1 (R&D Systems, Minneapolis, MN, USA) and 50 ng mL^−1^ for IL‐6 experiments (Miltenyi Biotec). In some cultures RA was also added at a final concentration of 1 nM (Sigma). Cultures were incubated at 37°C with 5% CO_2_ for at least 72 h before being analyzed for flow cytometry or sorted for Foxp3 expression. All conditions were set up in at least duplicate and repeated at least twice.

### T‐cell culture for Smad signaling analysis

Purified CD4^+^ T cells were isolated from Foxp3‐GFP BALB/c male mice as per method outlined above and washed in serum‐free media [Dulbecco’s modified Eagle’s medium containing 100 U mL^−1^ of penicillin and 100 μg mL^−1^ of streptomycin and 2 mM l‐glutamine (Thermo Fisher Scientific)]. The cells were then cultured at 2 × 10^6^ cells in 200 μL volume of serum‐free media in 5‐mL round‐bottom tubes that were loosely lidded (BD Biosciences, Franklin Lakes, NJ, USA) at 37°C with 5% CO_2_. The samples were left for at least 4 h in serum‐free media before 20 ng mL^−1^ TGF‐β or TGM was added for varying culture lengths between 16 h and 15 min in a reverse time course experiment, as specified in the figures.

### Staining for flow cytometry

Cells were washed in phosphate‐buffered saline (PBS) and stained for viability using FVS510 (BD Biosciences) which was diluted 1:1000 in PBS and 100 μL added to each sample of cells and left in the dark at room temperature for 15 min, then washed twice in FACS buffer (PBS containing 0.5% bovine serum albumin, 5 μM EDTA and 0.5% sodium azide). To reduce nonspecific binding, samples were incubated with 50 μL of polyclonal rat IgG (Sigma; diluted 1:50 in FACS buffer) for 15 min on ice and protected from light, then washed once in FACS buffer by spinning at 400*g* for 5 min and removing supernatant. Samples were then stained for flow cytometry by adding 50 μL of fluorescent antibodies in Brilliant Staining Buffer (BD Biosciences) at the concentrations as follows: anti‐CD3‐PE/y7 (clone 145‐2C11 at 1/200), anti‐CD4‐BB700 (clone RM4‐5 at 1/400), anti‐CD103‐BV785 (clone M290 at 1/200), anti‐PDL1‐BV711 (clone MIH5 at 1/400), anti‐CD39‐BV421 (clone Y23‐1185 at 1/100), anti‐CD25‐BV421 (clone PC61 at 1/400), anti‐CD73‐PE (clone TY/23 at 1/200), anti‐CD45.2‐BV711 (clone 104 at 1/200, BD Biosciences) and anti‐CD126‐PE/Cy7 (clone D7715A7 at 1/100, BioLegend, San Diego, CA, USA).

Samples that were also stained for intracellular antigens were fixed and permeabilized using Foxp3 Transcription Factor Buffer kit (Invitrogen, Waltham, MA, USA) and stained with antibodies in perm/wash buffer as follows: anti‐Foxp3‐ef450 (clone FJK‐16s, eBioscience, San Diego, CA, USA, 1/100) and anti‐ROR‐γt‐PE (clone AFJKS‐9, eBioscience, 1/100). Samples were then washed prior to analysis on the BD Celesta (BD Biosciences).

### Cell barcoding and Phosflow

Samples that were assayed for SMAD signaling analysis were fixed by adding 2 mL of prewarmed 1× Lyse/Fix buffer (BD Biosciences) at the end of the experiment, and mixed by inverting five times and then leaving in a 37°C water bath for 10–12 min. The tubes were then spun at 600*g* for 6 min to wash the cells, and then vortexed thoroughly before adding another 2 mL of FACS buffer and repeating the wash steps a further two times. The cells were then permeabilized by thoroughly pipetting 1 mL ice‐cold Perm Buffer III (BD Bioscience) and leaving samples on ice for 30 min. The cells were then washed by adding 3 mL of FACS buffer and spinning at 600*g* for 6 min and then vortexed thoroughly. The cells were resuspended in 500 μL of 50% Perm III (BD Biosciences) diluted in PBS and 60 μL of Violet Fluorescent Cell Barcoding (BD Biosciences) solution reconstituted as per manufacturer’s instructions and left in the fridge in the dark for 30 min. The cells were then resuspended in 3 mL of FACS buffer and wash steps were repeated a further two times and all barcoded samples were then pooled together and washed by spinning at 600*g* for 6 min. The cells were then resuspended in 100 μL of FACS buffer and Phosflow antibody mix containing anti‐mouse CD3‐BV785, anti‐mouse CD25‐BV650, anti‐mouse Foxp3‐AF488, anti‐mouse CD4‐BB700, anti‐Smad 1(pS463/pS465)/Smad 8 (pS465/pS467) and anti‐Smad 2 (pS465/pSS467)/3 (pS423/pS425), all purchased from BD Biosciences and used at the manufacturers’ recommended concentrations and added in 1 mL of FACS buffer and left at room temperature in the dark for 1 h. The cells were then washed three times in FACS buffer and resuspended in 1 mL prior to flow analysis.

### Fluorescent cell sorting

Naïve CD4^+^ T cells were isolated from the spleens of naïve Foxp3‐GFP BALB/c, Foxp3‐GFP C57BL/6 or IL‐10‐GFP‐Foxp3‐RFP C57BL/6 transgenic mice using the autoMACS protocol as described above, and for *in vivo* and TSDR experiments further sorted using the BD Aria III sorter (BD Biosciences). Cells that were purified by cell sorting prior to Treg induction were stained with anti‐CD4‐Percp/cy5.5 (clone GK1.5, BioLegend) and anti‐CD25‐APC (clone PC61, eBioscience), and cells which were CD4^+^CD25^–^Foxp3‐GFP/RFP^–^ were sorted into 15‐mL falcon tubes (BD Biosciences) containing 700 μL FBS. After sorting cells were washed three times in culture media and used as the starting population for Treg induction. After 4 days of Treg culture as described above, cells were sorted using Foxp3 reporter (either GFP or RFP‐expressing Foxp3 mice) and sorted into PBS containing 10% FBS to a purity of at least 98% using BD AriaIII (BD Biosciences). Prior to injection or culture, cells were washed three times in PBS or culture media depending on the purpose.

### ImageStream analysis

Purified CD4^+^ T cells were cultured in serum‐free media as above (section) overnight and stimulated with 20 ng mL^−1^ TGM or TGF‐β for 1 or 4 h. Then, the samples were fixed with 200 μL of 4% paraformaldehyde (PFA) solution in PBS (Sigma) to a final concentration of 2% PFA and left in a 37°C water bath for 20 min. The samples were transferred into a 1.5‐mL Eppendorf tube, spun at 600*g* for 6 min and then washed twice in PBS. After the final wash the cells were resuspended in 100 μL perm wash solution (1× PBS with 0.1% Triton X‐100) containing 0.2 μg anti‐Smad 2/3 AF488 (Santa Cruz Biotechnology, Dallas, TX, USA) and equivalent rat‐IgG antibody (Sigma) to block nonspecific binding, and samples were left in the fridge in the dark for 40 min. Cells were then washed in 1 mL of FACS buffer twice before being resuspended in 50 μL of PBS containing 0.5 μm DRAQ5 (BioLegend). Samples were run on the Amnis ImageStream X MKII and analyzed using the IDEAS software (Amnis, Seattle, WA, USA). Cells were subsequently gated on focused cells, single cells, double‐positive (Smad 2/3‐AF488 and DRAQ5 positive) and nuclear localization was calculated using the similarity dilate score between the 2 markers, with those having a higher score indicating the Smad2/3 is nuclear localized.

### Cytokine bead array of IL‐17 responses in EAE

Splenocytes from immunized EAE mice were processed into a single‐cell suspension as described above. The cells were cultured with 30 μg mL^−1^ of the immunizing protein pMOG_35–55_ (GenScript, Piscataway, NJ, USA) at a concentration of 1 × 10^6^ cells per well in a 96‐well plate and cultured for 72 h at 37°C in 5% CO_2_. The supernatant was removed and immediately stored at −80°C until cytokine bead array analysis. IL‐17A was analyzed using the mouse IL‐17A Flex Set cytokine bead array as per manufacturer’s instructions (BD Biosciences).

### Treg‐specific demethylation region

Naïve CD4^+^ T cells were isolated from the spleens of naïve Foxp3‐GFP BALB/c mice using the autoMACS protocol as described above, and further sorted using the AriaIII sorter (BD Biosciences). Cells that were purified by cell sorting prior to Treg induction were stained with anti‐CD4‐Percp/cy5.5 (clone GK1.5, BioLegend) and anti‐CD25‐APC (clone PC61, eBioscience), whereas cells which were CD4^+^CD25^–^Foxp3‐GFP^–^ were sorted into 15‐mL Falcon tubes (BD Biosciences) containing 700 μL FBS. After sorting cells were washed three times in culture media and used as the starting population for Treg induction. After 4 days of Treg culture as described above, cells were sorted using Foxp3‐GFP reporter (and sorted into PBS containing 10% FBS to a purity of at least 98% using BD AriaIII (BD Biosciences). The sorted cells were then plated into 24‐well plates with αCD3/αCD28 beads (Miltenyi Biotec) at a concentration of 1:2 (beads to cells) with additional IL‐2 (1000 U mL^–1^, Miltenyi Biotec) and 20 ng mL^−1^ of either TGF‐β or *Hp‐*TGM. At day 12 post reculture the cells were sorted again for Foxp3‐GFP on the BD AriaIII (BD Biosciences) and 20 000 cells were collected in Eppendorf tubes. The cells were then pelleted by centrifugation for 10 min at 500*g* and supernatants removed completely. The cell pellets were stored at −80°C until TSDR analysis was performed. Genomic DNA from cells of interest was obtained using the NucleoSpin Tissue kit (MACHEREY‐NAGEL, Düren, Germany). Genomic DNA was subjected to bisulfite conversion using the EZ DNA Methylation Kit (Zymo Research, Irvine, CA, USA). The murine TSDR was amplified by PCR containing 100 ng of bisulfite‐converted genomic DNA, HotStarTaq PCR buffer (Qiagen, Hilden, Germany), 1 U HotStarTaq DNA polymerase, 2.5 mM MgCl_2_ and 0.38 µM each of TSDR‐for (AAGGGGGTTTTAATATTTATGAGG) and TSDR‐rev (CCTAAACTTAACCAAATTTTTCTACCA) primer in a final volume of 25 µL (cycle: 95°C for 15 min; 45 × 95°C for 30 s, 57°C for 1 min, 72°C for 1 min; 72°C for 7 min). The PCR product was analyzed by gel electrophoresis. The pyrosequencing procedure was performed on a PyroMark Q96 ID (Qiagen) according to the manufacturer’s protocol, including 40 µL of the PCR product, PyroMark Gold Q96 reagents (Qiagen), PyroMark buffers (Qiagen), Streptavidin Sepharose (GE Healthcare, Chicago, IL, USA) and the sequencing primers TSDR1 (AACCAAATTTTTCTACCATTA), TSDR2 (AAAACAAATAATCTACCCC) or TSDR3 (AATAAACCCAAATAAAATAATATAAAT). The methylation rate was determined by the PyroMark Q96 software (Qiagen). A rate was excluded if the quality criteria (PyroMark Q96 standard settings) failed for that CpG motif.

### Analysis of gene expression of iTregs by NanoString analysis

Isolated splenocytes from male BALB/c Foxp3‐GFP mice were processed into a single‐cell suspension. CD4^+^ cells were further selected for using the autoMACS pro (Miltenyi Biotec) and a CD4 T‐cell isolation kit (Miltenyi Biotec). MACS‐purified cells were then cultured in T25 vented flasks with an anti‐CD3/anti‐CD28 bead‐to‐cell ratio of 1:2, 400 U mL^–1^ IL‐2 (Miltenyi Biotec) and 40 ng mL^–1^ TGM or TGF‐β for 3 days prior to FACS sorting of samples for FoxP3^+^ cells and FoxP3^–^ cells. Naïve CD4^+^ T cells and nTregs samples were prepared and FACS sorted (not cultured prior to NanoString analysis).

RNA lysates of cells were obtained by resuspending 10 000–20 000 of sorted cells per μL of RLT buffer (Qiagen). Samples were immediately frozen at −80°C until analysis. On the day of analysis, 1.5 μL of cell lysate was directly analyzed using a NanoString mouse immunology panel (V1) and run on a nCounter FLEX analyzer as per the manufacturer’s instructions (NanoString, Seattle, WA, USA). Hybridization was performed for 18 h at 65°C and samples were processed using the NanoString prep station set on high sensitivity. Images were analyzed at maximum (555 fields of view). Data were normalized using nSolver 4.0 software (NanoString).

### Experimental autoimmune encephalomyelitis experiments

Female C57BL/6 mice (8–10 weeks of age) were obtained from the University of Edinburgh and immunized for EAE as previously described.^42^ In brief, mice were injected subcutaneously in the hind flanks with 50 μg MOG_35–55_ peptide (GenScript) in complete Freund’s adjuvant (Sigma) supplemented with *Mycobacterium tuberculosis* (500 μg/mouse, BD Biosciences). Each mouse was also administered 200 ng of pertussis toxin (Sigma) intraperitoneally on days 0 and 2. Mice were weighed daily from the onset of disease and scored as follows: 0, normal; 1, partial tail paralysis; 2, full tail paralysis; 3, paralysis in one hind limb; 4, paralysis in both hind limbs and 5, moribund. Mice which reached a weight loss of greater than 20% or a score of 5 for longer than 24 h were euthanized. Mice received 1 × 10^6^ Tregs (sorted CD4^+^CD25^+^Foxp3‐GFP^+^ cells from C57BL/6 mice which were induced *in vitro* using either TGM or TGF‐β) by intravenous injection in 200 μL PBS 2 days prior to EAE induction.

### Dextran sulfate sodium (DSS)‐induced colitis

Colitis was induced in male C57BL/6 congenic mice (CD45.1) by administering 2% DSS (36 000–50 000 MW, MP Biomedicals, Santa Ana, CA, USA) in the drinking water *ad libitum*. In addition, mice received 1 × 10^6^ iTregs (sorted CD4^+^CD25^+^Foxp3‐GFP^+^ cells from C57BL/6 mice which were induced *in vitro* using either *Hp‐*TGM or TGF‐β) 3 days prior to DSS commencement. Mice were weighed and scored daily using a disease activity index matrix comprising body weight, blood, stool consistency and general appearance to aid objective comparison of the clinical progression of disease. Scores for each parameter are out of 4 and summed to give a disease activity index out of a maximum of 16. Animals were euthanized if they lost greater than 20% of their original body weight and experiments were terminated at day 7 or 8, depending on disease severity, and mesenteric lymph nodes and spleens were isolated for analysis by flow cytometry.

### Statistics

Graphs and statistics were analyzed using Prism (GraphPad, San Diego, CA, USA). Data are presented as means ± standard error of the mean (s.e.m.). One‐way or two‐way ANOVA or *t‐*tests (paired or unpaired) were used where appropriate, with nonparametric tests being applied if data were not normally distributed. *P < 0.05, **P < 0.01, ***P < 0.001.

## Conflict of Interest

We declare no competing financial interests.

## AUTHOR CONTRIBUTIONS

**Madeleine White:** Conceptualization; Investigation; Methodology; Writing‐original draft. **Danielle Smyth:** Investigation; Methodology. **Laura Cook:** Methodology; Writing‐review & editing. **Steven Ziegler:** Conceptualization; Funding acquisition; Writing‐review & editing. **Megan K Levings** **:** Conceptualization; Funding acquisition; Writing‐review & editing. **Rick Maizels:** Conceptualization; Data curation; Formal analysis; Funding acquisition; Project administration; Supervision; Writing‐original draft; Writing‐review & editing.
