# Supplementary material for: The parasite cytokine mimic Hp‐TGM potently replicates the regulatory effects of TGF‐β on murine CD4+ T cells
Source: Immunol Cell Biol. 2021 Jul 1;99(8):848–64. doi: 10.1111/imcb.12479 (PMC9214624; doi:10.1111/imcb.12479)
Supplement: Supplementary file 1 [file IMCB-99-848-s001.pdf]

Supplementary figure 1

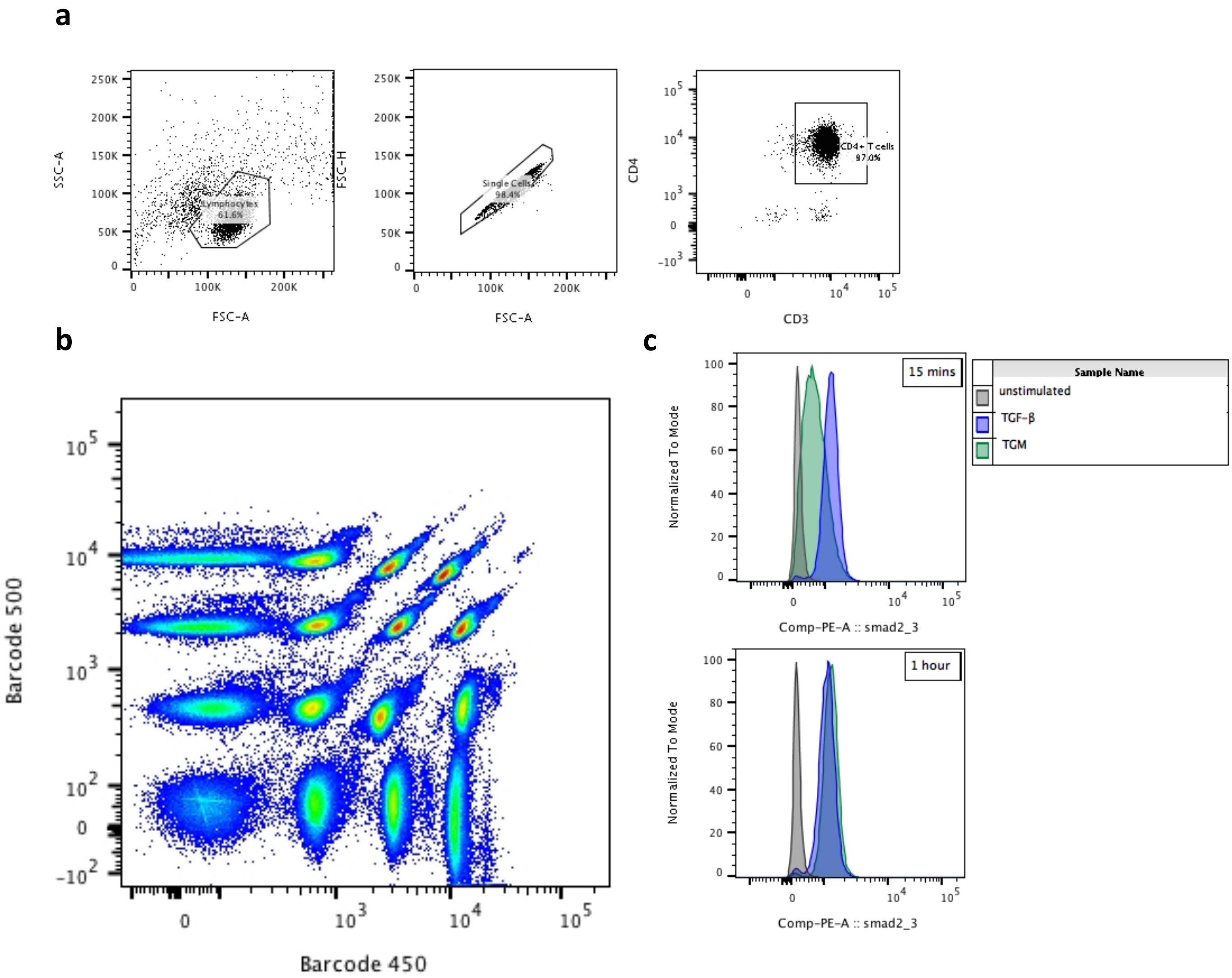

Supplementary figure 1.

CD4$^+$ T cells were isolated from the spleens of naïve Foxp3-GFP reporter BALB/c mice and cultured in serum-free media for 4 hours prior to the addition of *Hp*-TGM or TGF-β, in a reverse time-course beginning at 16 hours and ending with a 15-minute stimulation. (**a**) Gating strategy for lymphocytes, single cells and CD4$^+$CD3$^+$ T cells. (**b**) On CD4$^+$ T cells gated above, samples which were barcode labelled with varying concentrations of violet dye; these dyes are excited by the violet laser and emit in the 450nm or 500nm channel on the flow cytometer. The labels of 450 hi, 450 med, 450 low or 450 neg vs 500 hi, 500 med, 500 low or 500 neg staining allow for 16 different samples to be pooled prior to pSmad2/3 staining which increases confidence in small differences in MFI. (**c**) Histograms showing pSmad2/3 levels in samples at 15 minutes and 1 hour which shows the delay in pSmad2/3 activation with TGM.

Supplementary figure 2

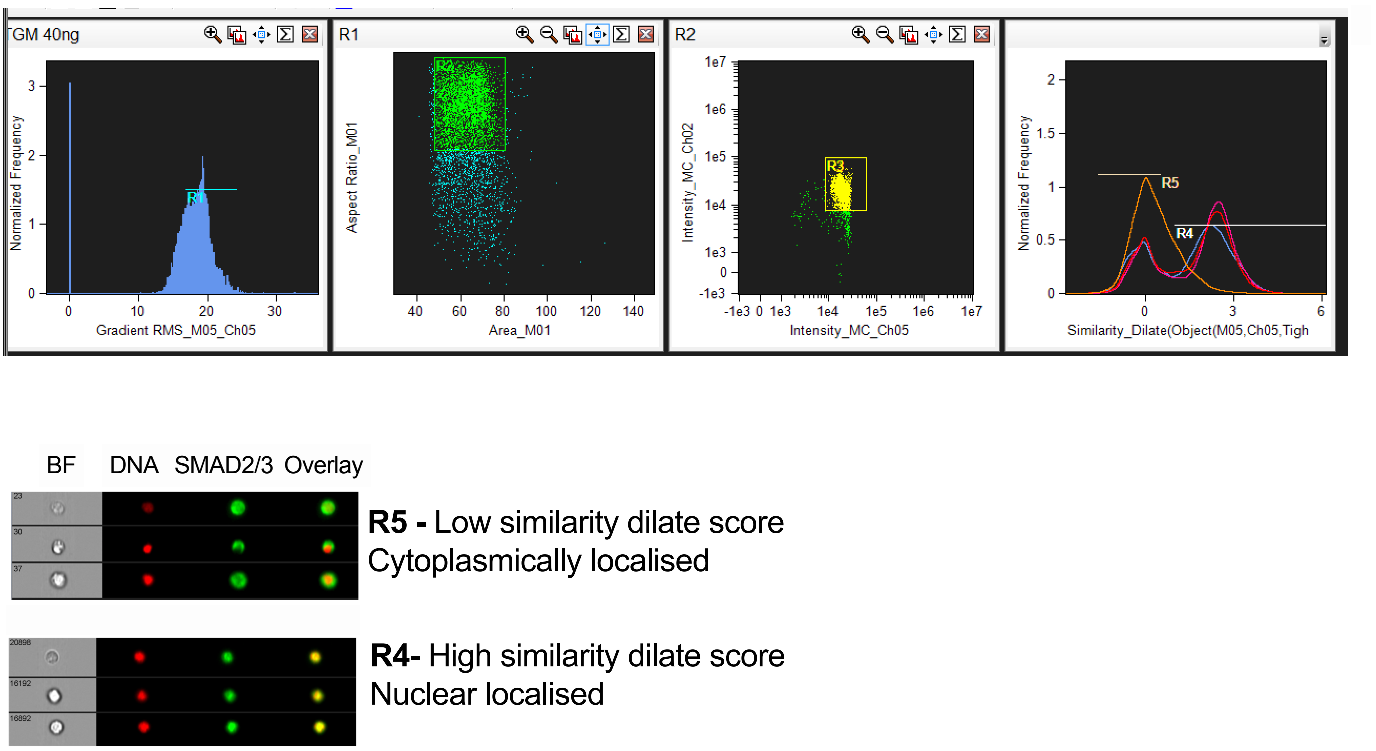

Supplementary figure 2.

CD4$^+$ T cells were isolated from naïve C57BL/6 mouse spleens and cultured in serum-free media for 12 hours prior to the addition of 20 ng.mL^{-1} of *Hp*-TGM or TGFβ. (**a**) Gating strategy for imagestream analysis. Gated on cells, focused cells, single cells and cells that are positive for both the markers of interest (DRAQ5 and Smad2/3). (**b**) Similarity Dilate score using the Nuclear Localisation analysis wizard in the Imagestream IDEA Software (Amnis Imagestream, Millipore). Samples with a low Similarity Dilate score around 0 where the Smad2/3 was found to be located in the cytoplasm, in contrast a high Similarity Dilate score indicated Smad2/3 was nuclear localised.

Supplementary figure 3

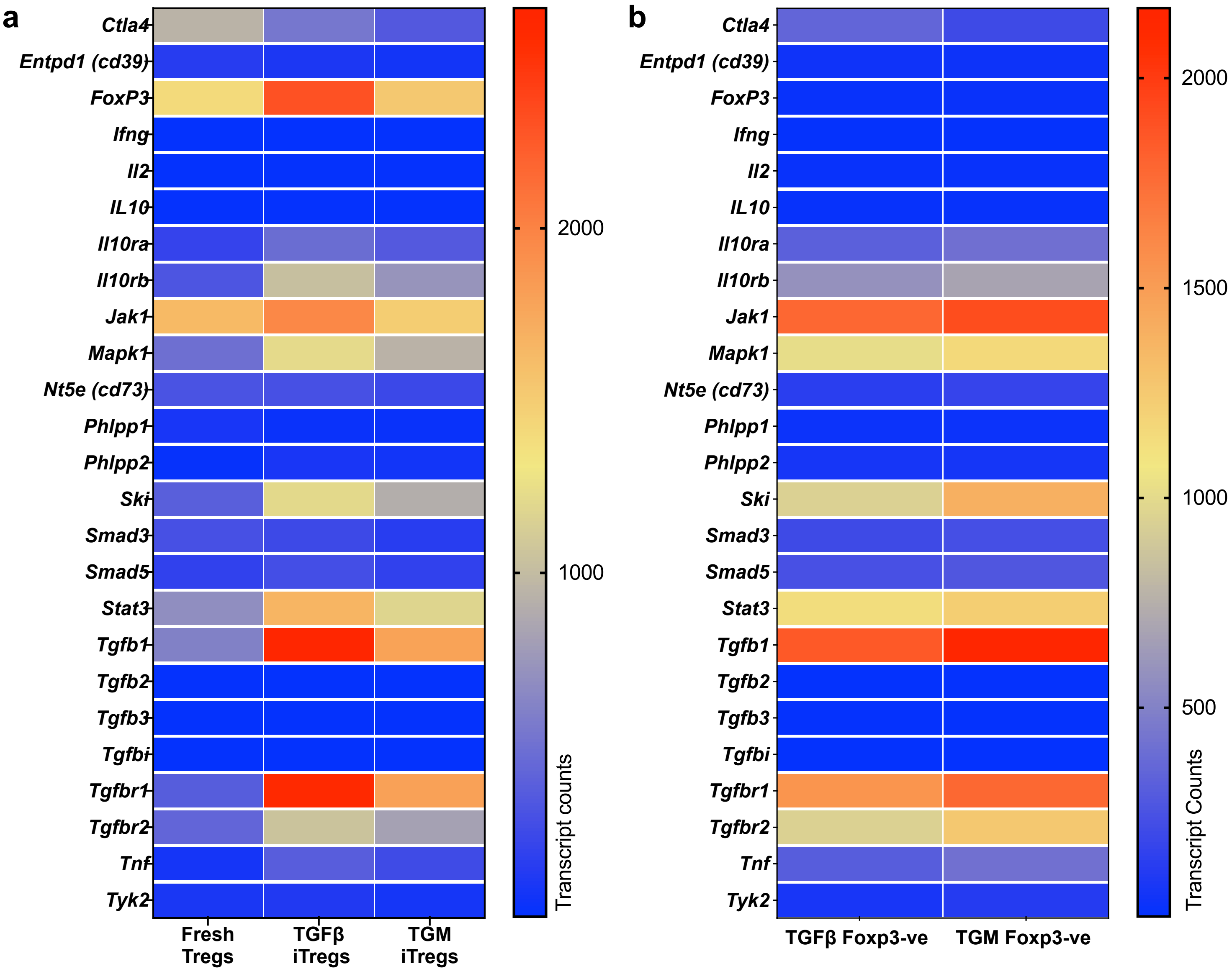

Supplementary figure 3.

CD4$^+$ T cells were isolated from the spleens of naïve Foxp3-GFP reporter BALB/c mice and cultured in the presence of anti-CD3/anti-CD28 beads, IL-2 and either *Hp*-TGM or TGF-β for 3 days. The cells were sorted based on Foxp3 expression and gene expression was analysed using the mouse immunology gene-set (V1) from NanoString. (**a**) Heatmap showing the gene expression of isolated CD4$^+$ T cells that were cultured in the presence of *Hp*-TGM or TGF-β for 72 hours prior to sorting and analysis in comparison with gene expression in already committed natural Tregs (nTregs) freshly isolated from the mouse. (**b**) Heatmap showing the gene expression of sorted Foxp3- cells from the same cultures in (**a**) after culture with *Hp*-TGM or TGF-β for 72 hours. Blue colour indicates a low level of gene expression and red colour indicates a high level of gene expression as determined by the number of transcript counts.

Supplementary figure 4

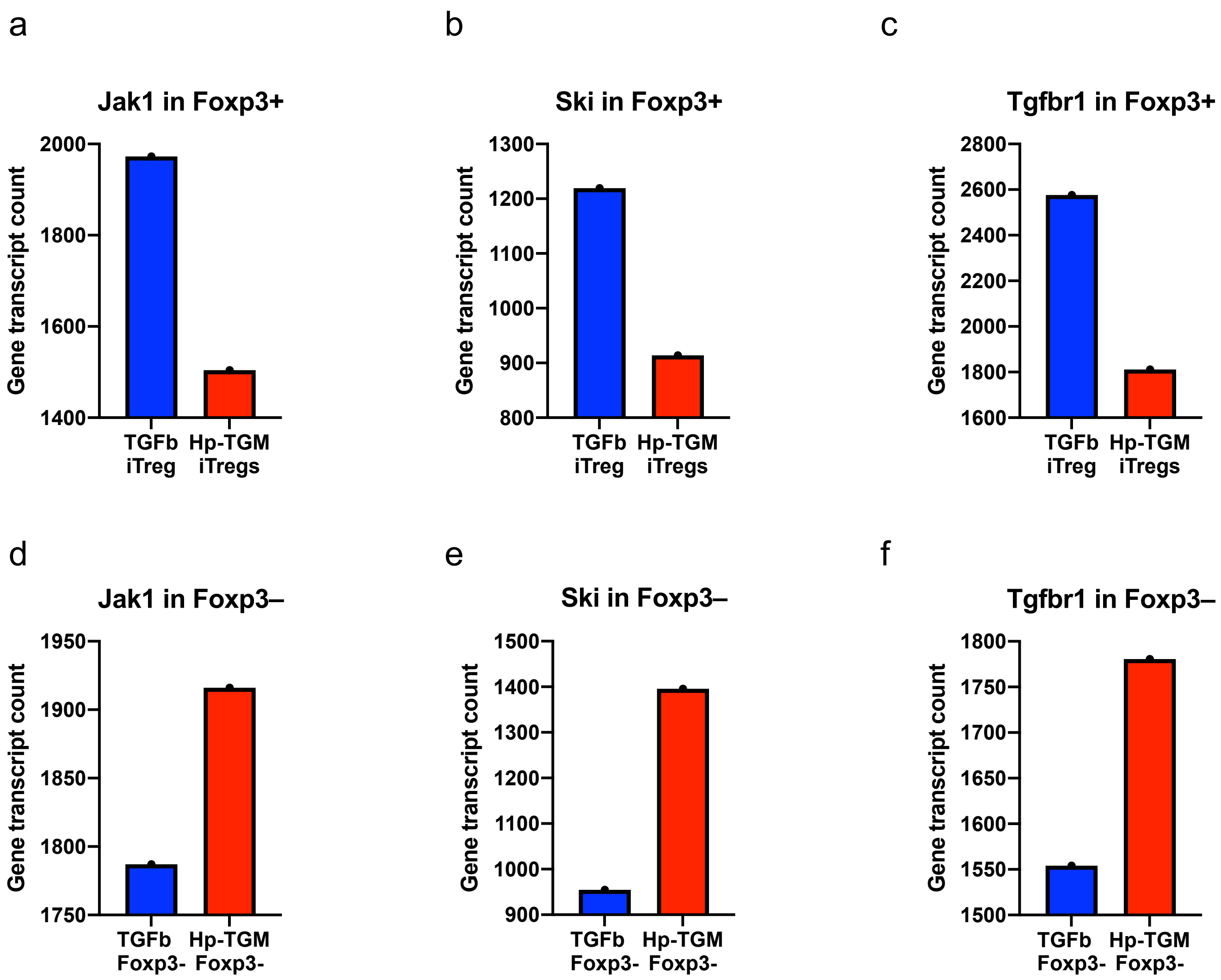

Supplementary figure 4

CD4$^+$ T cells were isolated from the spleens of naïve Foxp3-GFP reporter BALB/c mice and cultured in the presence of anti-CD3/anti-CD28 beads, IL-2 and either Hp-TGM or TGF-β for 3 days. The cells were sorted based on Foxp3-GFP and expression levels of *Jak1*, *Ski* and *Tgfbr1* in Foxp3+ (**a-c**) and Foxp3– (**d-f**) were analysed using the mouse immunology gene-set (V1) from NanoString.

Supplementary figure 5

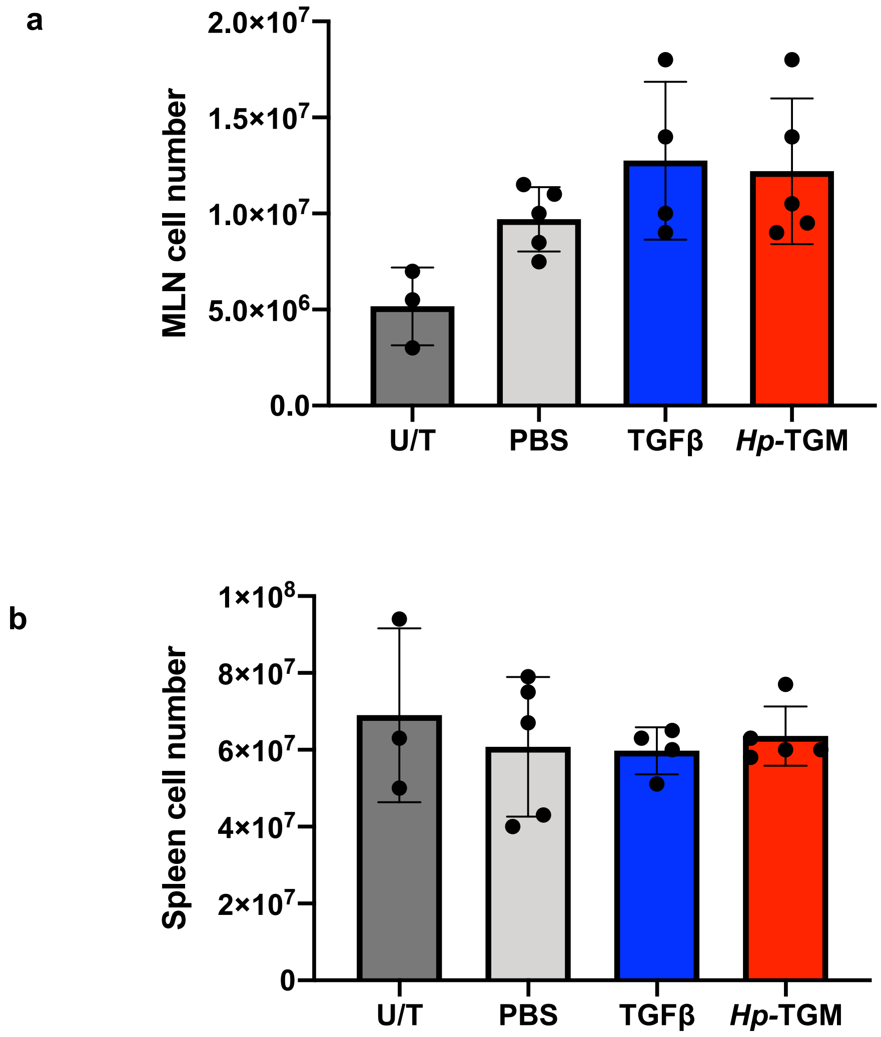

Supplementary figure 5.

Cell recoveries from mesenteric lymph nodes (**a**) and spleens (**b**) of mice receiving iTregs during DSS administration.
